# Catalpol promotes articular cartilage repair by enhancing the recruitment of endogenous mesenchymal stem cells

**DOI:** 10.1111/jcmm.18242

**Published:** 2024-03-20

**Authors:** Congzi Wu, Zhenyu Shi, Qinwen Ge, HuiHui Xu, Zhen Wu, Peijian Tong, Hongting Jin

**Affiliations:** ^1^ Institute of Orthopaedics and Traumatology of Zhejiang Province The First Affiliated Hospital of Zhejiang Chinese Medical University (Zhejiang Provincial Hospital of Chinese Medicine) Hangzhou Zhejiang China; ^2^ The First College of Clinical Medicine Zhejiang Chinese Medical University Hangzhou China; ^3^ Department of Orthopaedic Surgery The First Affiliated Hospital of Zhejiang Chinese Medical University Hangzhou China; ^4^ Department of Orthopaedic Surgery Tongde Hospital of Zhejiang Province Hangzhou China

**Keywords:** articular cartilage, cartilage defect, catalpol, chondrogenesis, stem cell homing

## Abstract

Articular cartilage defect is challenged by insufficient regenerative ability of cartilage. Catalpol (CA), the primary active component of Rehmanniae Radix, could exert protective effects against various diseases. However, the impact of CA on the treatment of articular cartilage injuries is still unclear. In this study, full‐thickness articular cartilage defect was induced in a mouse model via surgery. The animals were intraperitoneally injected with CA for 4 or 8 weeks. According to the results of macroscopic observation, micro‐computed tomography CT (μCT), histological and immunohistochemistry staining, CA treatment could promote mouse cartilage repair, resulting in cartilage regeneration, bone structure improvement and matrix anabolism. Specifically, an increase in the expression of CD90, the marker of mesenchymal stem cells (MSCs), in the cartilage was observed. In addition, we evaluated the migratory and chondrogenic effects of CA on MSCs. Different concentration of CA was added to C3H10 T1/2 cells. The results showed that CA enhanced cell migration and chondrogenesis without affecting proliferation. Collectively, our findings indicate that CA may be effective for the treatment of cartilage defects via stimulation of endogenous MSCs.

## 1 INTRODUCTION

Articular cartilage defects are common in the clinical condition that may cause pain and dysfunction.^1^ The therapy of cartilage injuries remains challenging because of the poor regeneration ability of cartilage due to its avascular and aneural character, which leads to poor differentiation and migration in chondrocytes for self‐healing.^2^ Articular cartilage belongs to hyaline cartilage and mainly consists of water (60%–85%), type II collagen (Col2), proteoglycans and chondrocytes (2%–5%).^3^ The occurring articular cartilage defects may lead to degenerative changes and the inevitable development of osteoarthritis (OA), a dominant cause of disability worldwide. Over the past decades, various clinical management, including microfracture (MF), autologous chondrocyte implantation (ACI), osteochondral allograft, etc., have been performed to repair cartilage.^4^, ^5^, ^6^ However, drawbacks of these strategies, like unsatisfactory long‐term effects, have restricted their application.^7^, ^8^

Due to the limitations of current approaches for treating cartilage defects, mesenchymal stem cells (MSCs)‐based therapies have gained significant attention.^9^ MSCs are derived from multiple sources, like adipose tissue, bone marrow and human urine. These MSCs have the advantage of having extraordinary potential for proliferation and chondrogenic differentiation. However, there are still significant obstacles in the way of developing stem cell therapies, including the high operation costs, complicated cell expansion procedures and uncontrolled differentiation of transplanted stem cells.^10^ In addition, the uses of MSCs are restricted to exogenous stem cell injections and this approach has several drawbacks, including a poor stem cell survival rate and rejection.^11^, ^12^ Therefore, cell homing‐based treatment by recruiting endogenous MSCs to the injury site and maintaining chondrogenesis has recently offered a new way for tissue regeneration in situ.^13^, ^14^ The primary and widely recognized treatment, known as MF, is intended to recruit endogenous mesenchymal stem cells (MSCs) from the bone marrow for articular cartilage repair.^15^, ^16^ However, Recent evidence suggests that the clinical results of MF remain unsatisfactory, with abnormal fibrocartilage repair, because of the limited ability of MF in recruitment and chondrogenic differentiation of MSCs.^17^, ^18^ Therefore, bioactive components that can attract, activate and guide endogenous MSCs for normal hyaline cartilage regeneration emerge as a promising solution.

Traditional Chinese medicine (TCM) is a treasure trove of potential therapies and has been used to prevent cartilage degeneration for several centuries.^19^, ^20^, ^21^ One example is *Rehmanniae Radix*, which has catalpol (CA, C_15_H_22_O_10_; molecular weight, 362.33) as its primary active component. CA has many biological activities, including antioxidative, anti‐inflammatory and anti‐ischaemic effects, and exerts protective effects against various diseases, such as osteoporosis and knee osteoarthritis.^2^, ^22^, ^23^, ^24^ In recent studies, catalpol was shown to modulate the physiological functions of MSCs in various ways, including improvement in MSCs survival and myocardial differentiation, enhancement of osteogenic differentiation and promotion of osteogenesis‐angiogenesis coupling.^25^, ^26^, ^27^, ^28^, ^29^ However, studies on the impact of active ingredients of medicinal herbs on the recruitment of MSCs and maintaining its chondrogenic phenotypes in cartilage are still limited, and whether CA is effective against cartilage defects remains unknown. Therefore, we conjectured that CA could promote the migration of MSCs to damaged cartilage and stimulate chondrogenesis in an animal model, eventually achieving cartilage repair.

Here, we intraperitoneally injected CA to the surgical articular cartilage defect model mice and evaluated its effects by macroscopical observation, micro‐computed tomography (μCT) analysis, cartilage‐specific staining, immunohistochemistry (IHC). After that, CA's migration effect on MSCs was evaluated both in vivo and in vitro. Further, CA has been added to C3H10 T1/2 cells, and Alcian blue staining was performed to assess in vitro chondrogenesis. Finally, the chondrogenic effect of CA was confirmed by examining the mRNA level of chondrogenic differentiation‐related markers.

## 2 METHODS

### 2.1 Materials

**FIGURE 1:**
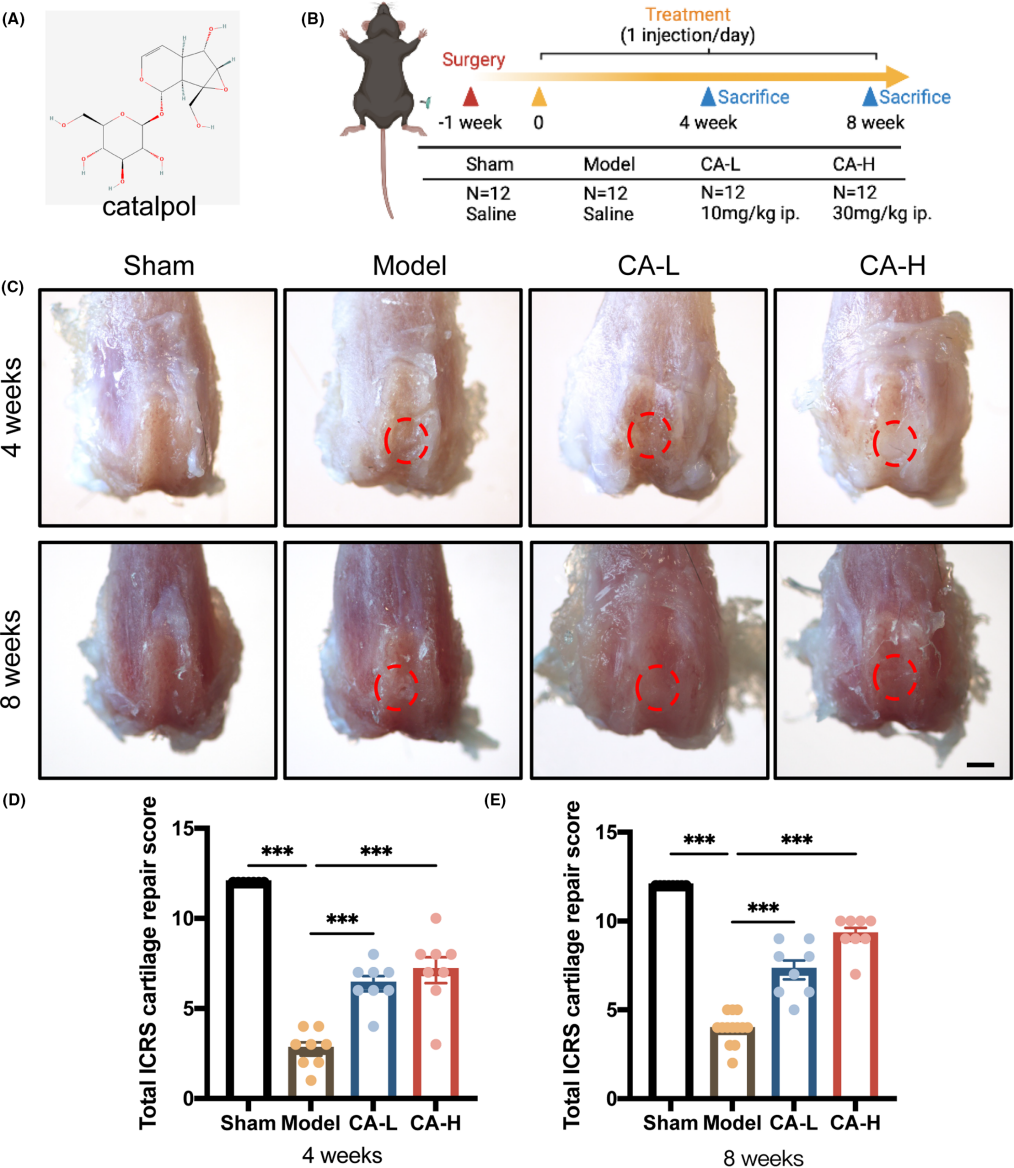
Study design and gross evaluations of repaired cartilage. (A) The chemical structure of Catalpol (CA); (B) Schematic design of the project; (C) Representative gross images of repaired cartilage at 4 and 8 weeks in the red circles. Scale bar, 1 mm. ICRS macroscopic scores of repaired cartilage at 4 (D) and 8 weeks (E) (*n* = 8). Data presented as means ± SEM by one‐way ANOVA with Turkey's post hoc test. ***p < 0.001.

Catalpol (CAS No. 327‐97‐9) was purchased from Chengdu Must Bio‐Technology Co., Ltd (Chengdu, China) (Figure 1A). Dulbecco's modified Eagle medium (DMEM), Dulbecco's modified Eagle's medium/F12 (DME/F12) and insulin‐transferrinselenous acid (ITS) were purchased from Gibco (New York, USA). Fetal bovine serum (FBS) and phosphate‐buffered solution (PBS) were bought from HyClone (Beijing, China). The Enhanced Cell Counting Kit‐8 (CCK‐8) was purchased from Bioss (Beijing, China). Crystal violet staining solution was purchased from Beyotime Biotechnology (Shanghai, China). Alcian‐Blue staining was purchased from Wuhan Goodbio Technology CO., Ltd (Wuhan, China). Anti‐Collagen II antibody (Col2) was purchased from Abcam (ab34712, 1:200). Anti‐CD90 was purchased from Proteintech (66766‐1‐Ig,1:300). The second goat anti‐rabbit antibody was purchased from the Invitrogen Corporation (MD, USA).

### 2.2 Animal care and CA administration

Ten‐week‐old C57BL/6J male mice were obtained from the Shanghai Laboratory Animal Co., Ltd. (Shanghai, China). All mice were housed at a constant room temperature of 23°C ± 2°C with a 12‐h light/dark cycle and free access to water and lab pellets. As reported previously, full‐thickness articular cartilage defects were created on bilateral knee joints.^30^ Briefly, after intraperitoneal injection with 0.3% pentobarbital sodium (50 mg/kg body weight), the mice's knee joint capsule was opened with the patellar dislocated laterally to expose the femur condyle fully. Then, a full‐thickness articular cartilage defect (0.45 mm diameter, 0.8 mm depth) was created on the trochlear groove using a custom‐made 26 G needle. The bleeding from the cartilage defect indicated the successful penetration of the subchondral bone.

In the sham group, mice were anaesthetised and subjected to the same surgical procedure without cartilage drilling. One week after the surgery, the mice were divided into four groups of six mice (*n* = 12 knees) per group for each timepoint: Sham group (Sham), model group (Model), low‐dose CA group (model mice treated with low‐dose CA, 10 mg/kg/day, ip.) and high‐dose CA group (model mice treated with high‐dose CA, 30 mg/kg/day, ip.) (Figure 1B).^24^, ^31^ We administrated CA intraperitoneally because CA could effectively target and locate cartilage defects with high bioavailability. All animal experiments complied with the ARRIVE guidelines, and the study was approved by the Experimental Animal Ethics Committee of Zhejiang Chinese Medical University (approval no. 202204‐0323).

### 2.3 Gross Observation and Micro‐computed tomography (μCT) analysis

At 4 and 8 weeks post‐surgery, the animals were sacrificed. The knee joints were collected and examined macroscopically using the International Cartilage Repair Society (ICRS) grading system.^32^ After being fixed with 4% paraformaldehyde (PFA) solution for 3 days, the samples were scanned at a high resolution of 9 μm with a μCT (Skyscan1176, Belgium). All parameters were set as a 45 kVp source and 500 mA current. NRecon v1.6 and CTAn v1.9 were used for three‐dimensional (3D) structure reconstruction and quantitative morphometric analysis, respectively. The partial images of the osteochondral defect regions (20 slices from the bottom up) were contoured for analysis. The bone volume fraction (BV/TV) was then calculated.

### 2.4 Histology

After fixed, mouse knee joints were decalcified in 14% EDTA (pH 7.4) for 14 days, embedded in paraffin and sectioned at 3 μm. The sections were subsequently stained with Alcian blue haematoxylin/Orange G (ABH/OG) and immunohistochemistry (IHC). The degree of repair for defective cartilage in the distal femur was scored by two blinded evaluators using the OARSI system.^33^ For IHC staining of Anti‐Collagen II antibody, all procedures were carried out according to the manufacturer's instructions. Briefly, slides were dewaxed and rehydrated before being soaked in sodium citrate solution for 4 h at 60°C for antigen retrieval. Then slides were incubated with primary antibodies at 4°C overnight, followed by binding with a secondary antibody (Invitrogen, 31234, 1:1000) for 20 min and stained in diaminobenzidine (ZSGB‐BIO) and counterstained with haematoxylin. For immunofluorescence (IF) of CD90, samples were incubated with a fluorescent secondary antibody (Thermo Fisher Scientific, A11001, 1:1000) for 40 min and counterstained with DAPI for 10 min. Images were captured with a microscope (Carl Zeiss, Göttingen, Germany) and semi‐quantified using ImageJ software.

### 2.5 Cell culture

C3H10 T1/2, a cell line with fibroblastic morphology and functionally similar to mesenchymal stem cells, was used in this study (ATCC, Manassas, VA, USA). C3H10 T1/2 were cultured in DMEM supplemented with 10% FBS at 37°C and 5% CO2. After reaching 80% confluence, cells were collected for further experiments.

### 2.6 Cell Counting Kit‐8 (CCK‐8) assay

The CCK‐8 assay was used to assess cell viability. C3H10 T1/2 cells (5.0 × 10^3^ cells/well) were seeded in 96‐well plates, and different CA concentrations ranging from 0 to 1600 μM were added to wells. After 24 or 48 h of incubation, the medium was replaced with 100 μL of fresh medium and 10 μL of CCK‐8 reagent for 2 h in the dark. The absorbance was measured on a microplate reader (Bio‐Rad, CA, United States) at 450 nm.

### 2.7 Wound healing assay

A wound healing assay was performed, as our previous study described.^34^ Briefly, C3H10 T1/2 cells were seeded in six‐well plates (1.0 × 10^5^ cells/well) and cultured with DMEM/F12 supplemented with 5% FBS. After reaching 80%–90% confluence, a wound was made using a sterile pipette tip. Afterwards, the cells were treated with different concentrations of CA for 0, 12 and 24 h. The images of migrations of C3H10 T1/2 cells into the scratch were captured using an inverted microscope (Olympus, Tokyo, Japan).

### 2.8 Transwell assay

The Transwell assay was used to evaluate cell migration. 5 × 10^4^ C3H10 T1/2 cells were seeded in the upper chamber of 24‐well plates (pore size, 8 μm; Corning, USA) with 200 μL serum‐free medium, while 500 μL medium containing 0, 100, 200,400 μM CA was added to the lower chamber. After 24 h incubation at 37°C, the upper chamber was fixed with 4% paraformaldehyde for 20 min. The cells on the bottom were stained with crystal violet solution for 30 min and the non‐migrating cells in upper chamber were removed with a cotton swab. The migrated cells were imaged and calculated.

### 2.9 Alcian blue staining

C3H10 T1/2 cells were seeded in 24‐well plates (1.0 × 10^4^ cells/well), and the medium was replaced with DMEM/F12 supplemented with 5% FBS and 1% ITS for chondrogenic differentiation. Different concentrations of CA were added to the medium to observe the effects on chondrogenic induction. The cell medium was changed every other day. At day 21, cells were stained with Alcian‐Blue staining for 30 min at room temperature.

### 2.10 Quantitative real‐time polymerase chain reaction (qRT‐PCR)

Total RNA was harvested from the distal femoral articular cartilage and three duplicated wells of C3H10 T1/2 cells using an RNeasy kit (Qiagen, Germany). Takara Reverse Transcription kit (Otsu, Japan) was used to reverse‐transcribe mRNA into complementary DNA. Following this, quantitative polymerase chain reaction (qPCR) was performed using a SYBR Premix EX TaqTM kit (Takara) with a QuantStudioTM 7 Flex Real‐Time PCR System (Thermo Fisher Scientific, Inc.) The primer sequences for the genes are listed in Table S1.

### 2.11 Statistical analysis

Statistical analysis was performed using Prism 9 software (GraphPad, San Diego, CA). Data are expressed as means ± SEM and analysed by one‐way or two‐way analysis of variance (ANOVA) with Dunnett's for multiple comparisons. The *p* value <0.05 was considered statistically significant.

## 3 RESULTS

### 3.1 Gross Evaluation of cartilage repair

Articular joint samples at 4 and 8 weeks post‐surgery were harvested for gross evaluation of cartilage repair. After surgery, knees in the model group started to show a visible cartilage defect, including surface depression and distinct boundaries, at 4 and 8 weeks, resulting in ICRS scores of 2.7 and 3.9 at these two time points, respectively (Figure 1C–E). Intraperitoneal injection of CA in low or high did significantly promote cartilage repair and improved the integration to the border zone of the repaired tissue. The ICRS score in CA‐L and CA‐H groups examined 4 and 8 weeks post‐surgery were significantly higher than those in the model group. The ICRS score was highest in the CA‐H group at both 4 weeks (7.1 ± 0.7) and 8 weeks (9.3 ± 0.5) after surgery, while there was no significant difference with low dose or high dose treatments at each time point.

### 3.2 CA attenuate the bone loss at the defect site

**FIGURE 2:**
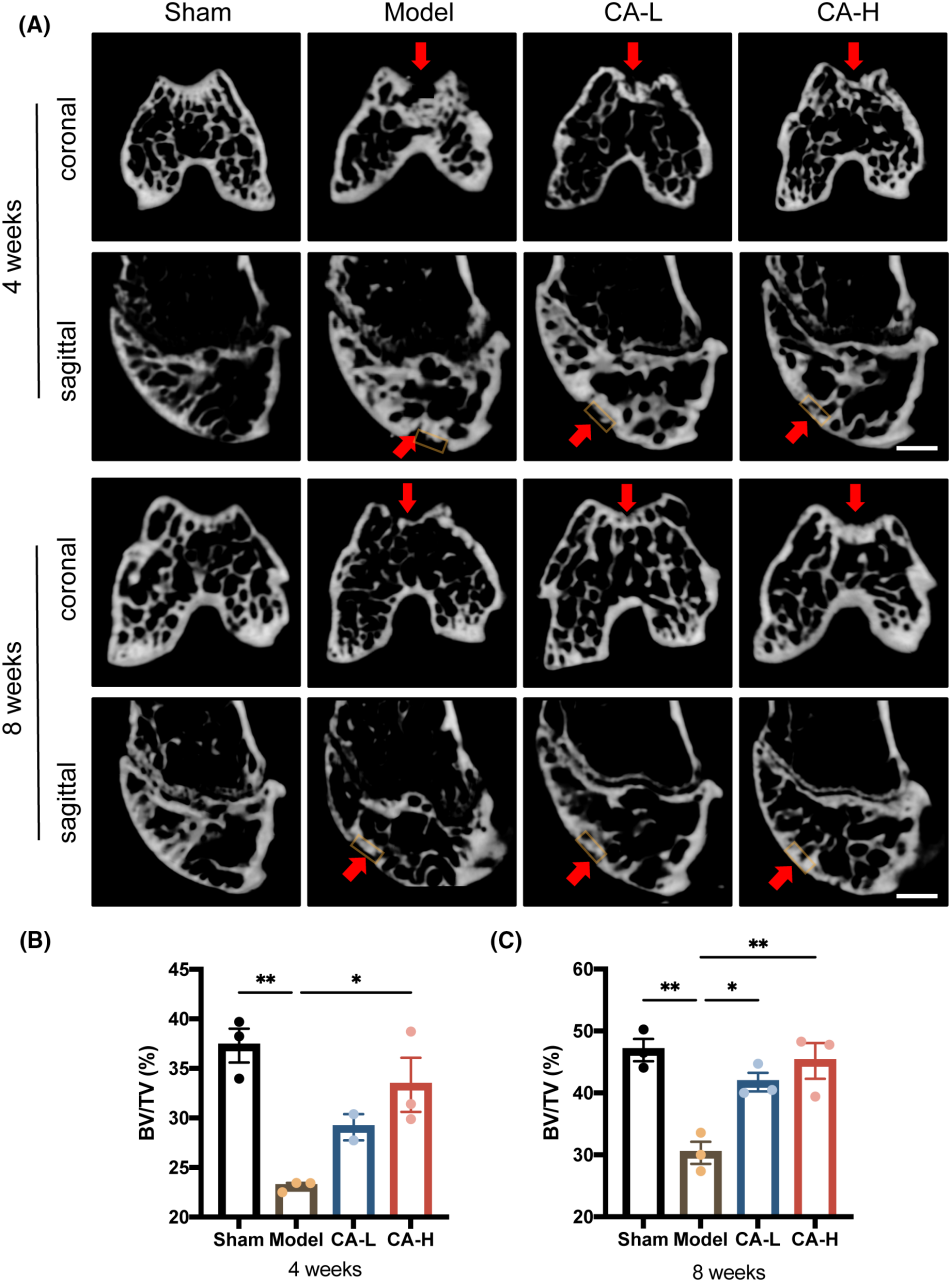
μCT image analysis of the defects at 4 and 8 weeks post‐surgery. (A) Representative coronal and sagittal images of different groups. Scale bar, 1 mm. The 3D standard microstructural analysis regions were indicated by orange rectangles and sites of the defect were indicated by red arrows. (B, C) Quantitative analysis of the BV/TV at the defect site (*n* = 3). Data presented as means ± SEM by one‐way ANOVA with Turkey's post hoc test. *p < 0.05 and **p < 0.01.

Regeneration of the bone and cartilage of the defective sites was graded according to the callus mineralized volume fraction (BV/TV) using μCT (Figure 2). Blank zones remained in the model group at 4 and 8 weeks after surgery. However, the defects in the CA‐treated group were filled over time, especially for the CA‐H group, which was covered by newly formed bone and cartilage‐like tissues, with smooth and continuous cartilage layers at both 4 and 8 weeks post‐surgery. Consistently, at 4 and 8 weeks after surgery, the BV/TV at the femoral defect site in the knee joint was significantly decreased in the model group compared to that in sham joints. However, this bone loss was not observed in the CA‐treated group. These data suggested that CA promotes bone and cartilage tissue repair at the initial time point of 4 weeks post‐surgery.

### 3.3 CA promotes cartilage repair at the defect site

**FIGURE 3:**
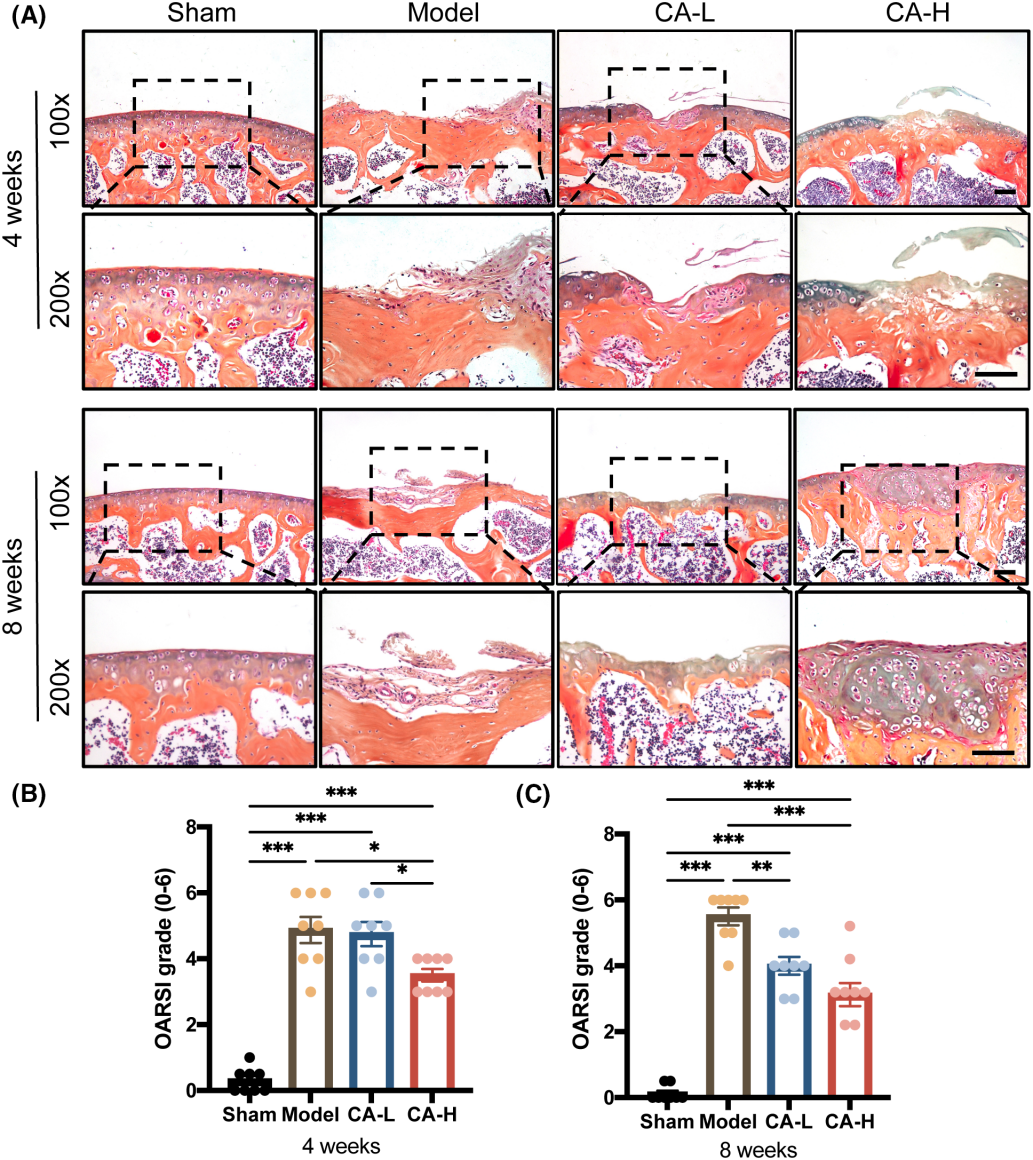
Histological assessment of repaired tissues at 4 and 8 weeks post‐surgery. (A) Representative ABH/OG staining images of different groups. Scale bar, 100 μm. (B, C) Quantitative analysis of the OARSI grade (*n* = 8). Data presented as means ± SEM by one‐way ANOVA with Turkey's post hoc test. *p < 0.05, **p < 0.01 and ***p < 0.001.

**FIGURE 4:**
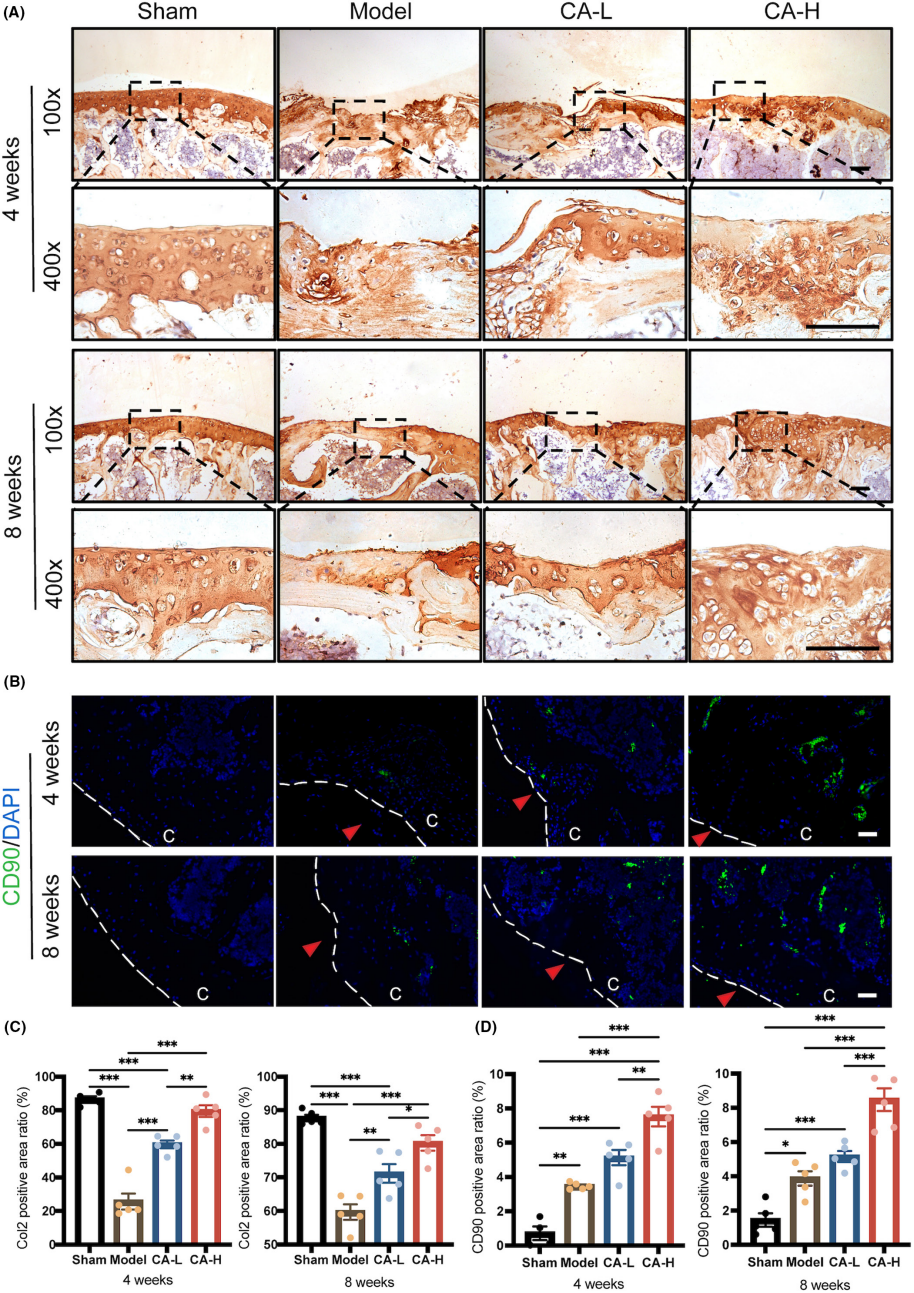
IHC staining of Col2 and in vivo recruitment of MSCs in repaired cartilages at 4‐ and 8‐weeks post‐surgery. (A) Immunohistochemistry staining and (C) semi‐quantitative analysis of the positive area ratio of Col2 of different groups. Scale bar, 100 μm. (B) Immunofluorescence staining (green) and (D) semi‐quantitative analysis of the positive area ratio of CD90 of different groups. The defect sites are indicated by red arrow. C, cartilage. Scale bar, 100 μm. Data presented as means ± SEM by one‐way ANOVA with Turkey's post hoc test. *p < 0.05, **p < 0.01 and ***p < 0.001.

ABH/OG staining was performed to investigate the morphology of articular cartilage (Figure 3). After surgery, the femoral defect site of the model group exhibited severe cartilage destruction up to the subchondral bone, including loss of proteoglycan and surface fibrillation at 4 weeks, and generated dense fibrous tissue at 8 weeks, resulting in OARSI scores of 4.9 and 5.5 at 4 and 8 weeks after surgery, respectively. On the contrary, the high‐dose CA could help regenerate the cartilage tissue with more abundant proteoglycan. Col2 is an indicator of matrix anabolism as well as hyaline cartilage. To evaluate the quality of repaired cartilage tissues, we analysed the expression of Col2 in articular cartilage by IHC staining (Figure 4A,C). Consistent with those of ABH/OG staining, the IHC results showed that lower Col2 expression was seen in the model group compared to the sham group, and high‐dose CA could inverse this trend, while low‐dose CA could partially enhance Col2 expression after surgery. The above results indicated that high‐dose CA could effectively repair mice cartilage injury with superior quality and quantity.

### 3.4 CA promotes migration and chondrogenesis differentiation of MSCs

**FIGURE 5:**
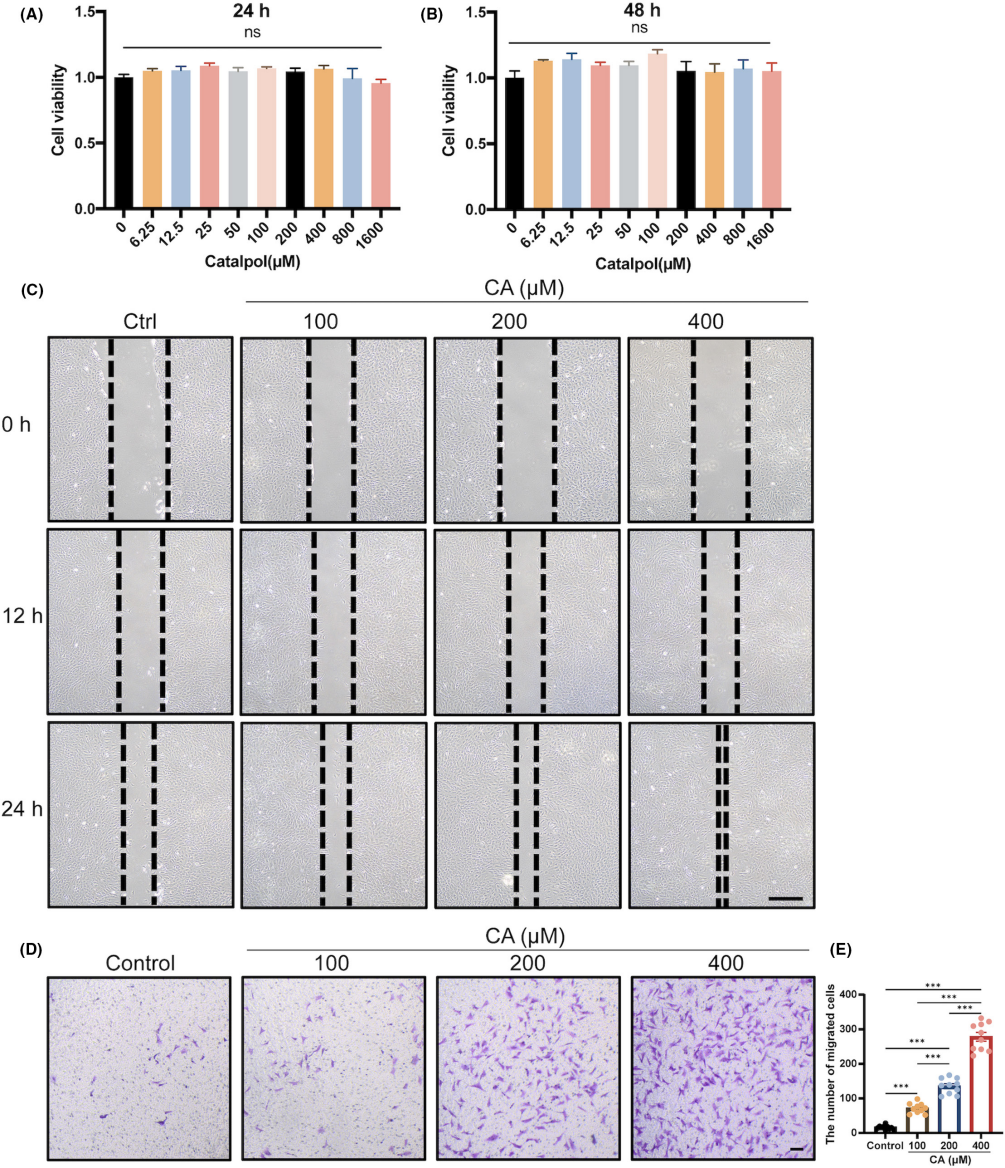
The impact of CA on MSCs viability and migration. (A, B) MSCs viability after 24 and 48 h of CA treatment at various concentrations. (C) Wound healing assay of MSCs at 0 h, 12 h and 24 h after CA treatment. Scale bar, 100 μm. (D, E) Transwell assay of MSCs stimulated by different concentrations of CA. Scale bar, 100 μm. Data presented as means ± SEM by one‐way ANOVA with Dunnett's or Turkey's post hoc test. *p < 0.05, **p < 0.01 and ***p < 0.001.

**FIGURE 6:**
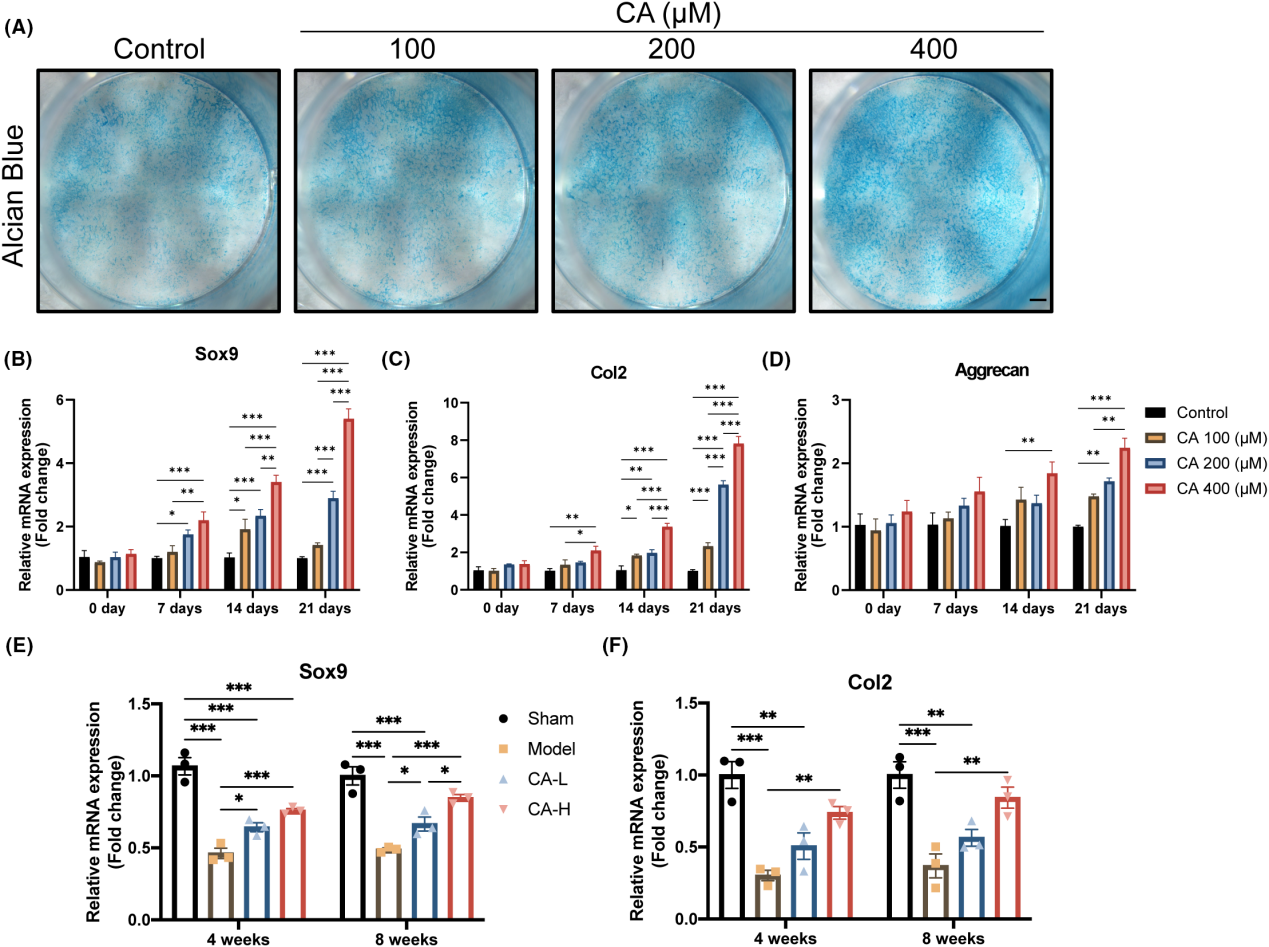
CA induces MSC recruitment and promotes chondrogenic differentiation. (A) Alcian blue staining of MSCs following chondrogenic differentiation with the treatment of CA. Scale bar, 1 mm. (B–D) The relative gene expression of MSCs following chondrogenic differentiation with treatment of CA for 0, 7, 14 and 21 days (*n* = 3). (E, F) The relative gene expression of repaired tissues at 4 and 8 weeks post‐surgery with the treatment of CA (*n* = 3). Data presented as means ± SEM by one‐way ANOVA with Turkey's post hoc test. *p < 0.05, **p < 0.01 and ***p < 0.001.

In situ, cartilage regenerating techniques heavily rely on MSC recruitment at wound sites. Thus, we identified CD90, a classical MSC marker, to confirm the in vivo impact of CA on the recruitment of MSCs (Figure 4B,D). almost no CD90 expression was detected in the sham group without a cartilage lesion. Only a small number of CD90+ cells were observed in the damaged sites of the model group, which may reflect that only very few MSCs migrated to the damaged sites in the absence of proper external stimuli. More CD90+ cells were observed in the CA group than in the model group, suggesting that CA has recruitment effect on MSCs. Subsequently, to verify the in vitro molecular mechanism by which CA‐induced cartilage repair, we examined MSCs activity, including migration, and chondrogenesis differentiation by CCK‐8 assay, wound healing assay, transwell assay, Alcian blue staining and qRT‐PCR. The CCK‐8 results showed that the addition of CA, unaffected by concentration, had no significant influence on cell proliferation (Figure 5A,B). In contrast, CA treatment significantly enhanced the migration of C3H10 T1/2 cells in a concentration‐dependent manner (Figure 5C–E). Furthermore, chondrogenic differentiation of C3H10 T1/2 cells was evaluated. The results of Alcian blue staining showed that CA had no significant effect on cartilage matrix synthesis after 7 and 14 days of chondrogenic differentiation. However, treatment with CA significantly stimulated cartilage matrix synthesis and accumulation in C3H10 T1/2 cells on 21 days (Figure 6A; Figure S1). RT‐qPCR results indicated that CA significantly improved the expression of Sox9, Col2 and Aggrecan genes after 7, 14 and 21 days of chondrogenic differentiation in vitro (Figure 6B–D). In addition, we found that mRNA expression of Sox9 and Col2 was reduced in the distal femoral articular cartilage of the model group. At the same time, CA treatment reversed these inhibitory effects of surgery in mice (Figure 6E,F). These results implied that CA could elevate MSCs migration and differentiation.

## 4 DISCUSSION

Growing evidence suggests that MSC homing is critical in treating tissue damage. In the present study, we investigate the efficacy of CA on cartilage repair in a mouse cartilage defect model induced by surgery and in C3H10 T1/2 cells. We found that CA could repair articular cartilage and contribute to subchondral bone defect filling, matrix anabolism and chondrophenotypic maintenance in vivo. To confirm the involvement of MSCs homing, we evaluated the migratory, and chondrogenic effects of CA on MSCs. We observed increased migration and chondrogenesis of MSCs due to CA treatment. Our findings indicate that CA substantially enhances articular cartilage repair, at least partly involving the promotion of chondrogenic differentiation of MSCs.

Because of various causes, such as cartilage degeneration, trauma and sports injury, articular cartilage defects are prevalent in clinical practice.^35^ Depending on the composition of the fibres, cartilage is divided into three types: hyaline cartilage, elastic cartilage and fibrocartilage. Articular cartilage belongs to hyaline cartilage, which plays supportive and lubricative roles in joints.^36^ In terms of injury and reparative approaches such as MF, the defect can be partially filled. However, the filled tissue comprises fibres or fibrocartilage, which lose some functions such as viscoelasticity and load bearing compared to hyaline cartilage, leading to OA or even worsening the symptoms.^37^ In our research, CA promoted cartilage repair and exhibited hyaline cartilage formation with higher Col2, indicating that CA benefits functional recovery of joints.

Growing evidence suggests that stem cell homing is critical in cartilage regeneration.^38^, ^39^, ^40^, ^41^, ^42^, ^43^ For example, Lei et al.^41^ non‐covalently incorporated PDGF‐BB and TGF‐β3 into microgels, which could recruit endogenous stem cells and have a therapeutic effect on cartilage damage. Furthermore, Murphy et al.^42^ co‐delivered BMP2 and soluble VEGFR1 following MF to skew differentiation of MF‐activated MSCs from fibrous tissues toward hyaline cartilage, as well as enhance stem cell homing to induce cartilage regeneration. In agreement with these studies, the data of IF, CCK‐8, wound healing assay and Alcien blue staining indicated that CA remarkably increased the migration and chondrogenesis of MSCs, suggesting CA could stimulate MSCs to generate cartilage for the treatment of localized chondral disease.

According to the results of μCT, subchondral bone mass was enhanced by CA administration. Consistently, Zhu et al.^26^ demonstrated that CA could significantly enhance the bone healing capacity of MSCs in rat bone defects and promote the differentiation of BMSCs into mature osteoblasts in vitro. Therefore, the enhanced osteogenic activity of MSCs by CA may have contributed to its bone regeneration.

## 5 CONCLUSIONS

In summary, we found that high‐dose CA contributes to cartilage regeneration, bone structure improvement and matrix anabolism increase via stimulation of endogenous MSCs, thereby promoting the repair of localized cartilage defects. The use of CA provides a potential treatment strategy for cartilage repair.

## AUTHOR CONTRIBUTIONS

**Congzi Wu:** Conceptualization (equal); data curation (equal); formal analysis (equal); writing – original draft (equal); writing – review and editing (equal). **Zhenyu Shi:** Conceptualization (equal); investigation (equal); methodology (equal). **Qinwen Ge:** Methodology (equal); validation (equal); writing – review and editing (equal). **Huihui Xu:** Methodology (equal); validation (equal); writing – review and editing (equal). **Zhen Wu:** Validation (equal); writing – review and editing (equal). **Peijian Tong:** Conceptualization (equal); funding acquisition (equal); resources (equal); writing – review and editing (equal). **Hongting Jin:** Conceptualization (equal); funding acquisition (equal); resources (equal); supervision (equal); writing – review and editing (equal).

## FUNDING INFORMATION

This research has been partially supported by the Natural Science Foundation of China (Grant nos. 82274280) and the Natural Science Foundation of Zhejiang Province (Grant No. LR23H270001). The funders had no role in study design, data collection and analysis, decision to publish or preparation of the manuscript.

## CONFLICT OF INTEREST STATEMENT

The authors have no conflicts of interest to declare.

## Supporting information


Figure S1

Table S1

## Data Availability

All relevant data are within the manuscript.
