# Supplementary material for: Catalpol promotes articular cartilage repair by enhancing the recruitment of endogenous mesenchymal stem cells
Source: J Cell Mol Med. 2024 Mar 20;28(7):e18242. doi: 10.1111/jcmm.18242 (PMC10955160; doi:10.1111/jcmm.18242)
Supplement: Supplementary file 1 — Figure S1 [file JCMM-28-e18242-s002.zip › Figure legends.docx]

Figure S1. Alcian blue staining of MSCs following chondrogenic differentiation with the treatment of CA For 0, 7, and 14 days. Scale bar, 1 mm.
