# Supplementary figures and images for: Catalpol promotes articular cartilage repair by enhancing the recruitment of endogenous mesenchymal stem cells
Source: J Cell Mol Med. 2024 Mar 20;28(7):e18242. doi: 10.1111/jcmm.18242 (PMC10955160; doi:10.1111/jcmm.18242)

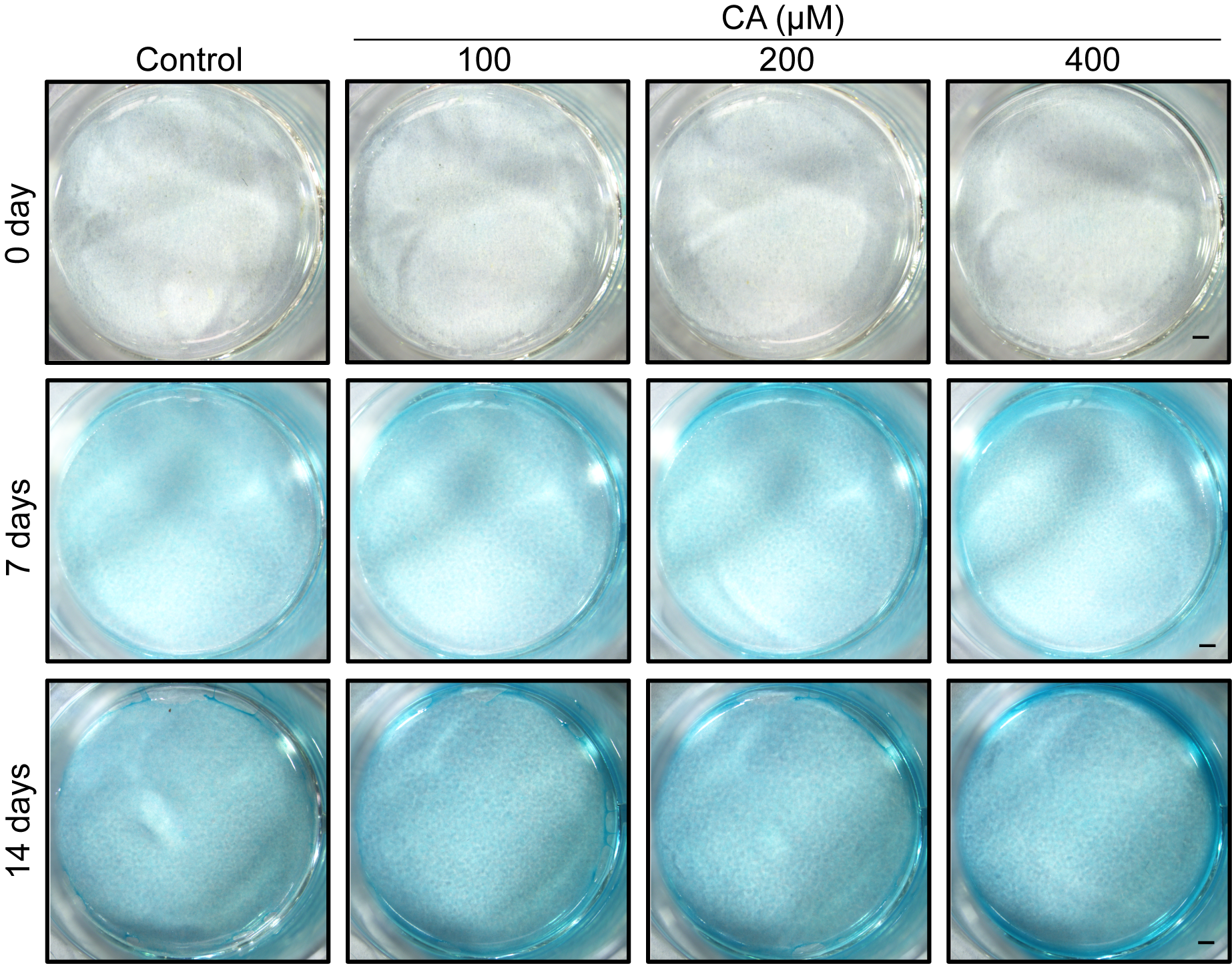

Supplement: Supplementary file 1 — Figure S1 [file JCMM-28-e18242-s002.zip › figS1 Alcien blue0-14day.tif]
