# Supplementary material for: Catalpol promotes articular cartilage repair by enhancing the recruitment of endogenous mesenchymal stem cells
Source: J Cell Mol Med. 2024 Mar 20;28(7):e18242. doi: 10.1111/jcmm.18242 (PMC10955160; doi:10.1111/jcmm.18242)
Supplement: Supplementary file 2 — Table S1 [file JCMM-28-e18242-s001.docx]

Table S1. Mouse real-time PCR primer sequences used in this study.

| Gene | Forward primer (5 ′ -3 ′) | Reverse primer (5 ′ -3 ′) |
| --- | --- | --- |
| SOX9 | CACACGTCAAGCGACCCATGAA | TCTTCTCGCTCTCGTTCAGCAG |
| Aggrecan | CAGCAAAGCAGACAGAACTAAG | AGAAAGGAACTGCTGGGATACG |
| Col2 | TGGTCCTCTGGGCATCTCAGGC | GGTGAACCTGCTGTTGCCCTCA |
| β-actin | CATTGCTGACAGGATGCAGAAGG | TGCTGGAAGGTGGACAGTGAGG |
